# Shear Wave Elastography of the Brachial Plexus and Target Muscle in Interscalene Block: A Narrative Review of Quantitative Tools for Patient-Centered Regional Anesthesia

**DOI:** 10.3390/jcm15135272

**Published:** 2026-07-06

**Authors:** Hye Joo Yun, Myokyung Choi, So Yeon Lee, Hyeonsook Jee

**Affiliations:** 1 Department of Anesthesiology and Pain Medicine, Eunpyeong St. Mary’s Hospital, College of Medicine, The Catholic University of Korea, Seoul 03312, Republic of Korea; soapdoll85@gmail.com (H.J.Y.);; 2 Department of Anesthesiology and Pain Medicine, Bucheon St. Mary’s Hospital, College of Medicine, The Catholic University of Korea, Bucheon 14647, Republic of Korea

**Keywords:** interscalene block, shear wave elastography, regional anesthesia, brachial plexus, deltoid muscle, ropivacaine, hemidiaphragmatic paresis, motor block, patient-reported outcomes, shoulder surgery

## Abstract

Ultrasound-guided interscalene block is a widely used analgesic technique for shoulder surgery, but its optimization is constrained by trade-offs between analgesic efficacy, motor impairment, and hemidiaphragmatic paresis. Shear wave elastography (SWE) quantifies tissue stiffness and has been proposed as a complementary, objective measurement that may help characterize the mechanical environment of nerves and muscles before and after local anesthetic injection. This narrative review synthesizes current evidence on SWE of the brachial plexus and deltoid muscle, with emphasis on technical considerations, reported reference values, and potential relevance to local anesthetic optimization in interscalene block. Available data show that SWE can quantify nerve and muscle mechanical properties, but direct clinical evidence in interscalene block remains limited; reproducibility at the cervical root level is variable; and, to date, no study has validated either brachial plexus SWE as a marker of intraoperative sensory blockade or deltoid SWE as a marker of regional anesthesia-induced motor block. We therefore propose a hypothesis-generating dual-target framework in which paired nerve and muscle SWE is investigated, alongside sensory testing, diaphragmatic assessment, and patient-reported recovery outcomes, as an exploratory surrogate of the local anatomical and mechanical changes that accompany the block, rather than as a direct readout of sensory or motor blockade itself. Standardized acquisition protocols, attention to floor effects in muscle measurements, and prospective validation studies are required before SWE can be considered a clinically actionable tool for patient-centered interscalene block.

## 1. Introduction

Shoulder surgery involving the glenohumeral joint and rotator cuff frequently relies on ultrasound-guided interscalene brachial plexus block (ISB) as the primary regional analgesic technique. Across randomized trials and observational studies, ISB has consistently lowered early postoperative pain scores and opioid requirements, resulting in a lighter early recovery burden than systemic analgesia alone [1,2,3,4,5]. The anatomical relationship between the brachial plexus and the phrenic nerve that underpins this efficacy also brings recognized risks, notably upper-limb motor weakness and hemidiaphragmatic paresis, particularly when traditional volumes and concentrations of long-acting local anesthetics are used [6,7]. Continuous interscalene catheter techniques have also been studied as a strategy to extend postoperative analgesia after arthroscopic rotator cuff repair [8].

Efforts to mitigate these complications have ranged from titrating local anesthetic volume, concentration, and total dose to substituting ISB with newer shoulder blocks that seek to preserve phrenic nerve function. Low-volume ISB protocols attenuate the incidence and severity of diaphragmatic dysfunction but cannot fully prevent it, and dose-finding as well as concentration-reduction trials indicate that, for selected indications, lower ropivacaine concentrations still provide clinically acceptable analgesia [9,10,11,12,13]. Disentangling the individual roles of concentration, volume, and total dose has proven challenging, largely because existing ISB trials tend to adjust several of these parameters concurrently rather than in isolation. Consequently, clinicians still lack clear, evidence-based guidance on how best to trade off analgesic efficacy against motor sparing, respiratory safety, and patient satisfaction when planning ISB for shoulder surgery.

A further limitation lies in how ISB has typically been studied. In most clinical trials, block onset and depth are inferred from simple bedside assessments—pinprick or cold sensation testing, ordinal motor scores, handgrip dynamometry, pain scales, and opioid use—rather than from direct tissue-level measurements [14]. These measures are unquestionably relevant to patients and clinicians, but they remain influenced by patient effort and observer judgment and do not directly report what is happening at the nerve and muscle level after local anesthetic injection. Standard B-mode ultrasound facilitates needle tracking and accurate injectate deposition yet offers no direct, quantitative measure of the physiological impact of the block itself [15].

Shear wave elastography (SWE) is an ultrasound-derived technique in which the velocity of shear waves generated within the tissue is used as a surrogate for tissue stiffness [16]. SWE has been used to characterize peripheral nerve mechanics in entrapment neuropathies and diverse neurological disorders and has been piloted for the quantitative assessment of deltoid muscle mechanical properties [17,18,19,20,21,22,23,24]. However, nerve-focused and muscle-focused SWE studies have rarely been integrated conceptually or methodologically, and almost none have been framed explicitly around patient-centered outcomes in regional anesthesia.

In this narrative review, we synthesize the available evidence on brachial plexus and deltoid SWE in the context of ISB and its trade-offs. Building on this synthesis, we propose a hypothesis-generating dual-target framework that couples nerve and target-muscle SWE with sensory testing, diaphragmatic assessment, and patient-reported recovery outcomes to support more patient-centered regional anesthesia. By emphasizing both mechanistic and patient-centered endpoints, we aim to provide a conceptual foundation for subsequent observational and interventional studies that seek to move beyond purely subjective block assessment.

## 2. Search Approach and Scope of This Narrative Review

This article is a narrative review supported by a structured literature search. The aim was interpretive synthesis rather than exhaustive evidence mapping; therefore, the review was not conducted as a formal systematic or scoping review [25], no protocol was registered, and no formal risk-of-bias tool was applied. We describe the search approach to enhance transparency and reproducibility of the narrative synthesis.

### 2.1. Literature Search

We searched PubMed/MEDLINE (National Library of Medicine/National Center for Biotechnology Information, National Institutes of Health, Bethesda, MD, USA; https://pubmed.ncbi.nlm.nih.gov/; accessed on 30 April 2026) for English-language publications from January 2000 through April 2026. Although the search window opened in 2000 to capture earlier work on the pharmacology of interscalene block and on hemidiaphragmatic paresis, shear wave elastography (SWE) itself was technically described in 2004 [26], and validated protocols for nerve and target-muscle SWE only became broadly available from approximately 2010 onward. Consequently, the substantive SWE literature integrated in this review is concentrated in the period from 2010 to 2026, with one online-first validation study published in 2025 and assigned to a 2026 journal issue [24] and additional 2024 work cited [27]. The search strategy combined terms related to shear wave elastography, sonoelastography, acoustic radiation force impulse imaging, the brachial plexus, cervical nerve roots, peripheral nerves, deltoid muscle, interscalene block, local anesthetic concentration, ropivacaine, diaphragmatic dysfunction, and phrenic nerve involvement. We also reviewed the reference lists of relevant original articles and reviews to identify additional eligible studies. No experimental instruments, agents, or statistical software were used by the authors in this narrative review; instruments and agents mentioned in the tables refer to the cited source studies rather than to original experiments performed by the authors.

### 2.2. Article Selection

We selected articles that provided clinically or methodologically relevant information on: (i) SWE of the brachial plexus, cervical nerve roots, upper-limb peripheral nerves, or the deltoid muscle; (ii) technical and interpretive considerations for nerve or muscle SWE; or (iii) ISB pharmacology, local anesthetic dose or concentration, diaphragmatic dysfunction, motor block, or patient-centered outcomes after shoulder surgery. Conference abstracts without full text, editorials without primary or methodological content, and non–peer-reviewed sources were not used as core evidence.

### 2.3. Narrative Synthesis

**Figure 1:**
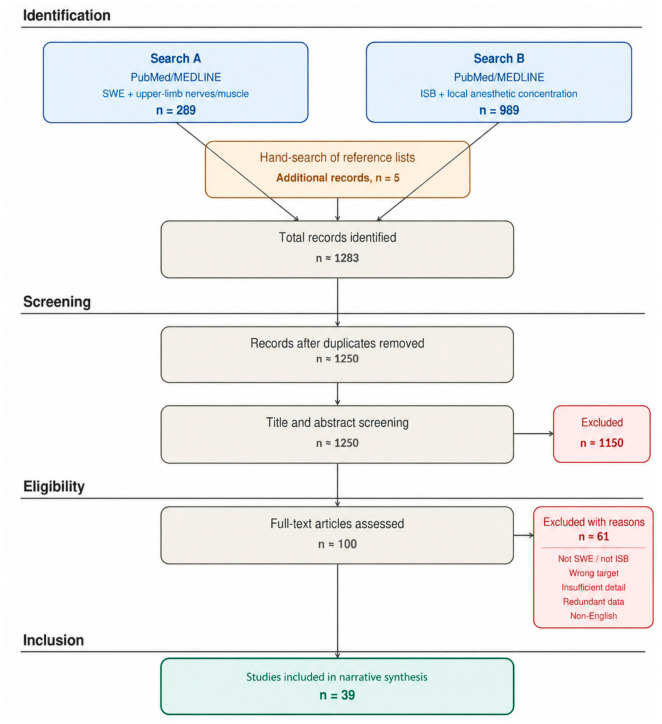
Structured literature search and article-selection pathway supporting this narrative review. Two parallel PubMed/MEDLINE search strategies were used to capture the two thematic strands integrated by the review: (i) shear wave elastography of upper-limb neural and musculoskeletal structures and (ii) interscalene brachial plexus block with local anesthetic considerations. Additional studies were identified by hand-searching reference lists. The numbers shown are provided for transparency and should be interpreted as descriptive rather than as a formal PRISMA-ScR flow for a systematic or scoping review [25]. Arrows indicate the flow of record identification, screening, eligibility assessment, and inclusion. Colored boxes are used only to visually distinguish the two parallel search strands, additional hand-searching, and the final narrative synthesis steps; they do not represent different levels of evidence or statistical categories. ISB, interscalene brachial plexus block; SWE, shear wave elastography.

We synthesized the included literature thematically across seven domains: SWE principles; brachial plexus reference values and reliability; factors affecting nerve stiffness; post-injection interpretation; deltoid SWE; local anesthetic dose and concentration trade-offs in ISB; and patient-centered research implications. For transparency, Figure 1 summarizes the structured search and article selection pathway that informed this narrative review; the reported record counts are descriptive and should not be interpreted as evidence of systematic completeness. Accordingly, we frame our conclusions according to the strength and directness of the available evidence rather than by applying a formal evidence grading system.

### 2.4. Use of AI-Assisted Tools

**Figure 2:**
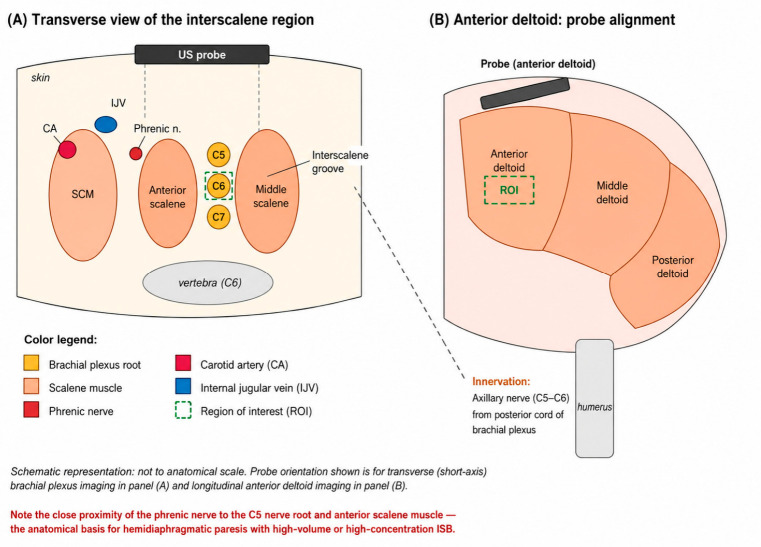
Schematic of anatomical structures relevant to SWE acquisition during interscalene block. (**A**) Transverse interscalene view showing the C5–C7 roots between the anterior and middle scalene muscles, with the adjacent phrenic nerve, carotid artery, internal jugular vein, and sternocleidomastoid. A representative region of interest on the C6 root is shown. (**B**) Deltoid segments and probe alignment for longitudinal anterior deltoid imaging. Drawings are schematic and not to anatomical scale. The dashed line indicates the conceptual linkage between the interscalene brachial plexus region in panel (**A**) and the target-muscle/innervation schematic in panel (**B**); it does not represent an anatomical structure.

**Figure 3:**
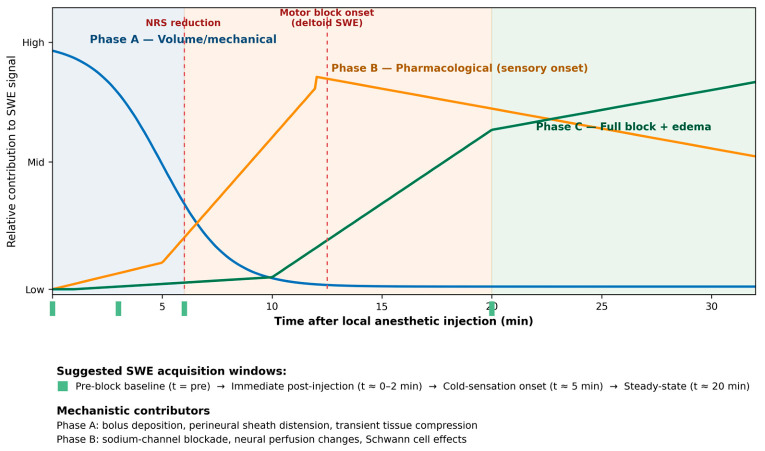
Hypothesis-generating time-resolved framework for post-injection brachial plexus SWE changes during interscalene block. Early measurements are likely dominated by volume-related mechanical loading; later measurements may reflect a combination of local anesthetic effects, tissue redistribution, sensorimotor block, and edema. Curves are conceptual and should not be interpreted as measured effect sizes. NRS, numerical rating scale; SWE, shear wave elastography.

**Figure 4:**
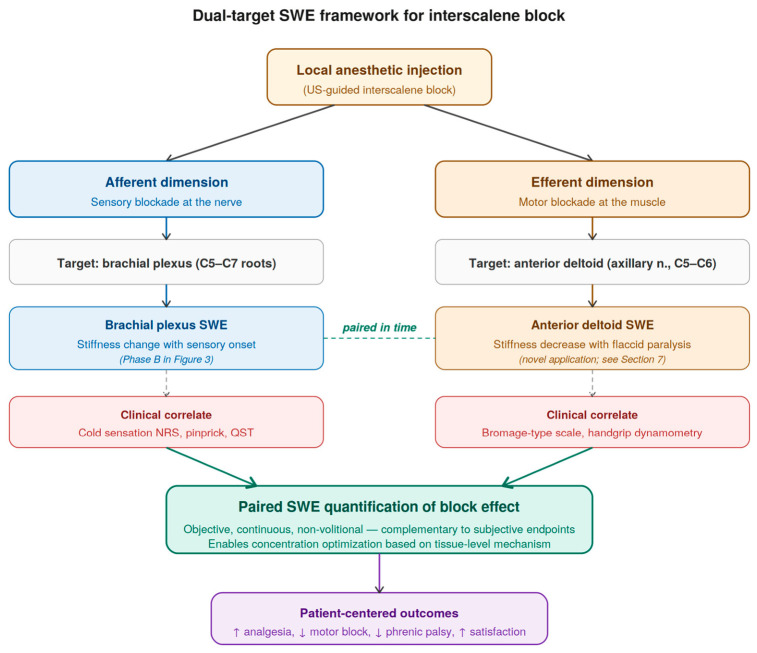
Proposed dual-target SWE framework for interscalene block research. Brachial plexus SWE and anterior deltoid SWE are positioned as exploratory surrogate measures of the local mechanical changes that accompany sensory and motor block, respectively, rather than as direct markers of conduction blockade itself. These measurements should be interpreted together with clinical sensory testing, diaphragm assessment, motor recovery, and patient-reported outcomes. Arrows indicate the conceptual flow from interscalene block-related local mechanical changes to quantitative SWE assessment and patient-centered outcomes. Colors are used only to visually distinguish anatomical targets, measurement domains, and outcome domains; they do not indicate statistical categories or levels of evidence. NRS, numerical rating scale; QST, quantitative sensory testing; SWE, shear wave elastography.

**Figure 5:**
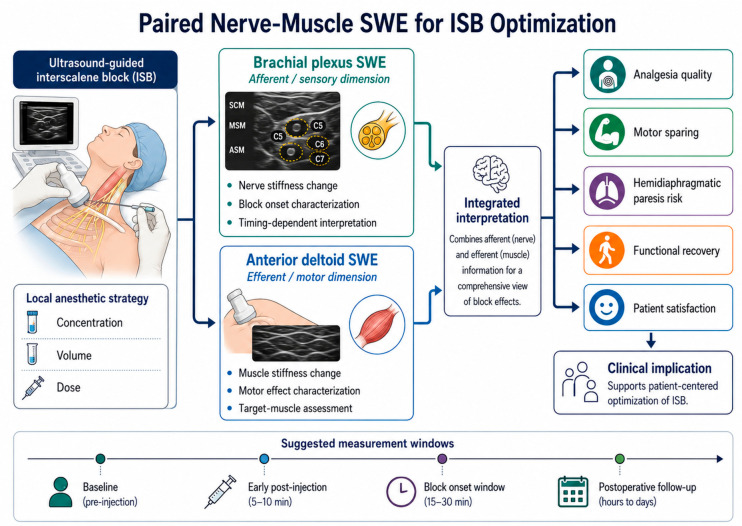
Central illustration of the proposed role of paired nerve-muscle shear wave elastography (SWE) in interscalene block (ISB) optimization. Local anesthetic concentration, volume, and total dose influence both nerve-level and target-muscle effects. Brachial plexus SWE is proposed as an exploratory afferent/sensory-domain marker, whereas anterior deltoid SWE is proposed as an exploratory efferent/motor-domain marker. When acquired at standardized time points and interpreted alongside conventional sensory testing, motor assessment, diaphragmatic evaluation, and patient-reported outcomes, paired SWE may help characterize trade-offs among analgesia quality, motor sparing, hemidiaphragmatic paresis risk, functional recovery, and patient satisfaction. Arrows indicate the proposed conceptual relationships among local anesthetic dosing variables, nerve- and muscle-level SWE measurements, conventional clinical assessments, and patient-centered outcomes; they do not imply validated causal pathways. This framework is hypothesis-generating and requires prospective clinical validation before routine clinical adoption. ISB, interscalene brachial plexus block; SWE, shear wave elastography.

**Figure 6:**
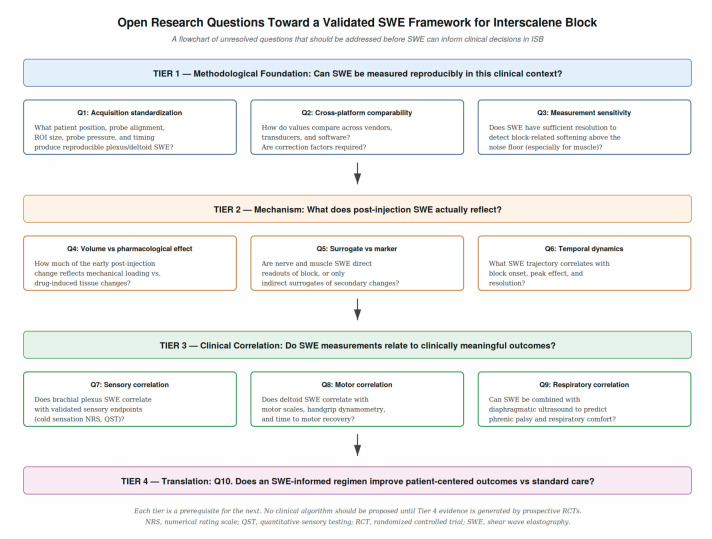
Flowchart of open research questions toward a validated SWE framework for interscalene block. The questions are organized into four sequential tiers that should be addressed before SWE can inform clinical decisions. Tier 1 (Methodological Foundation) asks whether SWE can be measured reproducibly in the regional anesthesia context. Tier 2 (Mechanism) asks what post-injection SWE signals actually reflect, distinguishing volume-related mechanical loading, pharmacological effects, and secondary tissue changes. Tier 3 (Clinical Correlation) asks whether SWE measurements correlate with established sensory, motor, and respiratory endpoints. Tier 4 (Translation) asks whether SWE-informed local anesthetic regimens improve patient-centered outcomes compared with standard care. Each tier is a prerequisite for the next, and no clinical algorithm should be proposed until prospective Tier 4 evidence is generated by randomized controlled trials. NRS, numerical rating scale; QST, quantitative sensory testing; RCT, randomized controlled trial; ISB, interscalene brachial plexus block; SWE, shear wave elastography.

We used artificial intelligence (AI)–assisted tools, specifically Claude Sonnet 4.6 and Claude Opus 4.6 (Anthropic PBC, San Francisco, CA, USA; https://www.anthropic.com/; accessed on 23 June 2026), for two specific and limited purposes: (1) language refinement of author-drafted prose, including grammar correction and improving clarity of expression; and (2) drafting of schematic illustrations for Figure 1, Figure 2, Figure 3, Figure 4, Figure 5 and Figure 6 as conceptual frameworks. All AI outputs were manually reviewed, edited, and approved by the authors. No AI tool was used to generate, analyze, or interpret primary data, conduct the literature search, select references, formulate scientific conclusions, or make clinical recommendations. The authors verified all citations, numerical data, and clinical claims against the original published sources. The authors accept full responsibility for the accuracy and integrity of all content in the submitted work.

## 3. Principles of Shear Wave Elastography and Interpretation in Neural and Muscular Tissues

### 3.1. Physical Basis

Unlike conventional gray-scale ultrasound, which depicts tissue echogenicity, SWE estimates a mechanical property of tissue from the speed at which shear waves propagate through it. Acoustic radiation force impulses produce small tissue displacements, and the resulting transverse waves travel faster through stiffer tissue [16]. Commercial systems report either shear wave velocity (m/s) or a derived Young’s modulus (kPa). The conversion to Young’s modulus assumes tissue homogeneity, isotropy, incompressibility, linear elasticity, and constant density; these assumptions hold only approximately in nerves and skeletal muscle [16,26].

### 3.2. Application to Peripheral Nerves and Skeletal Muscle

In nerves and skeletal muscle, the idealized conditions behind the Young’s modulus equation hold only loosely, and the largest source of deviation is anisotropy. Because both tissues are built from parallel fibers, a shear wave travels faster along the fiber axis than across it, so the same structure yields different readings depending on probe orientation [26,28]. At the brachial plexus, aligning the probe with the long axis of the nerve trunk tends to give higher and more repeatable values than a transverse approach, though between-study scatter remains substantial [20,21,29]. The deltoid adds a further wrinkle: its anterior, middle, and posterior portions run at different angles, so a protocol must fix the probe relative to each segment’s pennation to keep anisotropy from masquerading as a real stiffness difference [22,24].

A separate caveat concerns what the number actually represents. Bench work on excised nerve has shown that the tension applied to the tissue predicts wave speed better than the tissue’s own intrinsic stiffness does, implying that any SWE reading blends the material itself with whatever mechanical load it happens to be under at that moment [30]. Posture, joint angle, and the pull of neighboring soft tissue can therefore shift the value without any change in the nerve itself. At the interscalene level specifically, head rotation, arm abduction, and a supine-versus-seated change in position have each been shown to alter plexus readings [27,28]—which is precisely why identical positioning before and after the block is non-negotiable for any valid comparison.

### 3.3. Practical Considerations for ISB Research

Several recurring pitfalls in published brachial plexus and deltoid protocols bear directly on study design. The ROI should be kept small and seated consistently within the target, clear of vessels, fascial planes, and bone, all of which corrupt the reading [20,31]. Probe pressure matters as much as placement: even the gentle force of a resting hand compresses superficial tissue and inflates the measured stiffness, an effect that immersion or stand-off gel-pad techniques can blunt [28,32]. Finally, reproducibility at this site is not a given—reported inter-observer agreement for the brachial plexus ranges only from poor to moderate [21]—so trained operators, a fixed acquisition protocol, and averaging across repeated captures are needed to obtain usable data. Each of these constraints shapes how an ISB study using SWE should be built and how its results ought to be read.

## 4. Normative Shear Wave Elastography Values of the Brachial Plexus

### 4.1. Reference Values in Healthy Adults

Preliminary reference data for brachial plexus SWE at the interscalene level were reported by Bedewi et al., who examined 40 healthy adults [20]. Mean shear elastic modulus values were 16.9 kPa at C5, 15.7 kPa at C6, and 16.0 kPa at C7, with wide ranges across individuals [20]. These values suggest that cervical roots can be mechanically differentiated from adjacent scalene muscle, but absolute thresholds should be interpreted cautiously because equipment, probe orientation, ROI conventions, and patient positioning vary across studies [20,21,33]. The available reference values are summarized in Table 1.

### 4.2. Measurement Reliability in Brachial Plexus SWE 

How dependable these measurements are remains unsettled. Aslan et al., in a pilot study of healthy volunteers, found that intraclass correlation coefficients (ICCs) for shear wave and strain elastography alike sat only in the poor-to-moderate range, prompting them to doubt whether the technique is yet fit for routine plexus assessment [21]. The culprits they identified—ROI placement that depends on the operator, variable probe pressure, cervical roots that are small relative to the ROI, and inconsistent wave attenuation through the surrounding fascia—are largely anatomical. That interpretation is reinforced by the contrast with larger, more superficial nerves such as the median and tibial, where reliability is generally good to excellent [17,18]. The practical lesson for ISB work is that each patient should serve as their own control through paired pre- and post-block measurement, rather than leaning on population-level reference thresholds that the reliability data cannot yet support.

### 4.3. Pathological Comparisons and Threshold Values

Disease states do leave a detectable mark on plexus stiffness. In 32 patients with multiple sclerosis who had no clinical brachial plexopathy, Gürün et al. recorded higher mean elasticity and narrower root diameter than in 32 matched controls—a signature consistent with subclinical peripheral demyelination [33]. A comparable stiffening has been described in the plexus after breast irradiation, fitting the broader pattern in which neural fibrosis drives SWE values upward [34]. Taken together, these reports establish that SWE responds to structural change, but they also expose its main interpretive limit: the rise in stiffness is non-specific, carrying no signature unique to any one disease, and so must always be read against the clinical picture, cross-sectional imaging, and electrophysiology where available. The implication for ISB is direct—a stiffness shift seen after injection cannot simply be equated with one particular tissue process and demands careful mechanistic reasoning, the subject of the next section.

## 5. Factors Affecting Brachial Plexus Stiffness Relevant to ISB

### 5.1. Posture and Joint Position

Brachial plexus stiffness is sensitive to the mechanical loading of the upper limb and neck. Greening and Dilley demonstrated, using SWE in healthy volunteers, that neck contralateral flexion and shoulder abduction produced reproducible increases in median nerve and brachial plexus stiffness, attributable to longitudinal nerve excursion and increased intraneural strain [28]. Ciuffreda et al. extended these findings to the upper limb neurodynamic test sequences, showing that the order in which joint movements are imposed alters the magnitude and timing of stiffness changes in the median nerve and brachial plexus [27]. These observations have direct relevance for ISB: the patient’s head position, neck rotation, and shoulder posture during block performance and during pre- and post-block SWE measurement must be standardized, since inadvertent variation can produce stiffness changes that mimic or mask the effect of local anesthetic injection.

### 5.2. Patient-Related Factors

Demographic and anthropometric variables influence baseline brachial plexus stiffness to a modest but measurable extent. Bedewi et al. identified statistically significant sex differences at the C6 and C7 levels, although the magnitude of difference was small relative to inter-individual variability [20]. Age, body mass index, and handedness have not been consistently associated with brachial plexus stiffness in healthy adults, although reported sample sizes are small and statistical power for these subgroup analyses is limited [20,21]. In contrast, a clearer age-dependent trend has been documented for the deltoid muscle, particularly the anterior segment [24]. For clinical ISB studies, balancing or stratifying by sex and age remains prudent, especially when between-group comparisons are central to the study design.

### 5.3. Pathological Conditions Affecting Baseline Stiffness

Several disease states alter baseline brachial plexus stiffness and may therefore confound peri-block SWE interpretation. Multiple sclerosis [33], post-radiation fibrosis [34], diabetic polyneuropathy [17,18], and chronic compressive neuropathies [29] have all been associated with elevated nerve stiffness on SWE. Chronic shoulder pathology, including rotator cuff disease, has not been systematically evaluated for its effect on brachial plexus stiffness, but the high prevalence of these conditions among shoulder surgery candidates makes this a relevant consideration for ISB-related research [23]. Where feasible, pre-existing comorbidities affecting peripheral nerves should be recorded as covariates and either excluded or analyzed as planned subgroups.

### 5.4. Implications for Pre-Block Standardization

Taken together, these factors converge on a single methodological recommendation: SWE measurements before, during, and after ISB must be acquired under tightly controlled positioning conditions in patients whose baseline neural status is documented. Within-patient paired comparison—each patient serving as their own control—remains the most robust analytic approach, as it cancels out fixed baseline factors and isolates the contribution of the block itself [21,27]. This approach also aligns with the practical realities of perioperative imaging, where between-patient comparison at population scale is unlikely to be feasible. Table 2 summarizes the principal factors influencing brachial plexus stiffness on SWE and their relevance to ISB study design.

## 6. Mechanisms of Post-Injection Nerve Stiffness Changes

### 6.1. Direct Pharmacological Effects of Local Anesthetics

The primary pharmacological action of local anesthetics is blockade of voltage-gated sodium channels, but this mechanism does not in itself predict a change in tissue stiffness [35]. Any post-injection SWE change is likely to reflect a composite of mechanical loading, injectate spread, tissue hydration, vascular effects, and time-dependent drug-tissue interactions rather than a single pharmacological process. Consequently, post-block SWE should be interpreted as a quantitative adjunct to clinical assessment rather than as a direct surrogate for sodium-channel blockade.

### 6.2. Volume and Mechanical Effects of Injection

Before any drug effect registers, the injection itself changes the mechanics of the site. Delivering a bolus into the perineural sheath pushes tissue aside, swells the connective envelopes wrapped around the cervical roots, and resets the local state of tension. Lockwood and McLeod captured this directly: pairing B-mode with SWE during regional blocks, they saw 5 mL boluses alter both nerve dimensions and the mechanics of the tissue around them within seconds of delivery [31]. Since SWE responds to imposed stress and not solely to a tissue’s own stiffness [30], this mechanical disturbance should govern the earliest readings. The loading state then keeps evolving as the bolus tracks along fascial planes and spreads through the perineural space, so the recorded values may drift for some time before pharmacology contributes anything detectable.

### 6.3. A Time-Resolved Interpretive Framework

Because mechanical and pharmacological processes evolve on different time scales, acquisition timing is central to interpretation. Early measurements likely reflect mostly volume-related loading and local tissue displacement, whereas later measurements may include effects of drug distribution, sensorimotor block, and tissue edema. Future studies should therefore anchor SWE acquisition to clinically defined milestones, such as onset of cold sensation change, motor weakness, and diaphragmatic assessment, instead of relying only on arbitrary clock times.

### 6.4. Current State of Direct Evidence

Plausible as each pathway is, the direct evidence on how much, and in which direction, plexus stiffness actually moves after injection during ISB is thin. What exists is a handful of small, exploratory studies running heterogeneous protocols [19,31]. Perineural injection and local tissue displacement have been visualized elastographically in cadaver-to-patient translational work [36], yet no one has systematically mapped how concentration and time after injection jointly shape SWE values in the clinical setting. This is among the field’s most consequential blind spots, and closing it is a precondition for ever using SWE as a dependable readout of block onset or depth in everyday practice.

## 7. Shear Wave Elastography of the Anterior Deltoid: A Window into Motor Block Assessment

### 7.1. Methodological Foundations

The anterior deltoid is a plausible second target for SWE research in ISB. Anatomically, its motor supply is through the axillary nerve, with dominant C5–C6 contributions, and these roots are commonly affected by ISB [1]. Functionally, a motor block induced by regional anesthesia could alter passive muscle mechanical properties. However, this application has not been validated, and the deltoid should currently be considered a candidate research endpoint rather than an established clinical marker of block depth.

### 7.2. Normative Values and Influence of Age

Current reference figures come from Ivanov et al., who measured 40 healthy volunteers and reported mean stiffness of 23.2 ± 4.6 kPa anteriorly, 26.4 ± 5.6 kPa laterally, and 17.9 ± 5.2 kPa posteriorly [24]. Only the anterior segment showed a clear climb with age; the lateral and posterior portions were comparatively age-stable. For any ISB study that adopts the anterior deltoid as its motor readout, these two findings carry a practical warning: without stratifying by age and locking down probe orientation, ordinary anatomical variation could be mistaken for a block-induced change. These published deltoid reference values are summarized in Table 3.

### 7.3. Clinical Correlations in Shoulder Pathology

The clinical utility of deltoid SWE has been most extensively documented in shoulder arthroplasty. Schmalzl et al. reported that anterior deltoid stiffness measured by SWE correlated with patient-reported pain levels after reverse shoulder arthroplasty, supporting the use of SWE as a non-invasive correlate of clinically relevant muscle tension states [23]. Although the pathophysiological context of arthroplasty differs from that of regional anesthesia, these data demonstrate that deltoid SWE captures clinically meaningful variation in muscle mechanical state, a necessary condition for its use as a motor block marker.

### 7.4. Translation to Regional Anesthesia: The Gap

Although Saito et al. demonstrated that SWE can track muscle stiffness changes during anesthetic induction with opioids and neuromuscular blockers [37], no published study has yet validated deltoid SWE as a marker of motor block induced by brachial plexus regional anesthesia. The conceptual rationale is plausible: reduced neural drive and flaccid weakness may lower passive muscle stiffness, whereas pain, guarding, or residual tone may increase it. Prospective studies must therefore compare deltoid SWE with standardized motor testing, dynamometry when feasible, pain scores, and timing of recovery before deltoid SWE can be used as a clinically meaningful endpoint.

## 8. Local Anesthetic Concentration in Interscalene Block: Evidence and Patient-Centered Trade-Offs

### 8.1. Historical Trajectory: From High to Low Concentration

Earlier ISB practice commonly relied on relatively large volumes and high concentrations of bupivacaine or ropivacaine [11], achieving reliable analgesia but also producing frequent motor block and a high incidence of hemidiaphragmatic paresis [6,7]. Contemporary practice has shifted toward reducing the overall local anesthetic burden, but the evidence base is complicated by the fact that concentration, volume, and total dose are often modified together. This interdependence has made it difficult to attribute observed clinical effects to concentration alone.

### 8.2. Volume Reduction

Several randomized trials have focused primarily on reducing injectate volume while holding concentration constant. Riazi et al. showed that decreasing the volume of 0.5% ropivacaine from 20 mL to 5 mL preserved analgesia for shoulder arthroscopy while substantially lowering the incidence of diaphragmatic impairment [9]. Lee et al. similarly reported that 5 mL of 0.75% ropivacaine provided analgesia comparable to 10 mL but with less phrenic nerve paralysis [38]. Taken together, these studies support low-volume ISB as a plausible safety strategy, although residual phrenic involvement and respiratory compromise remain possible even with volumes as low as 5 mL.

### 8.3. Concentration Reduction

Concentration reduction is a complementary but methodologically more complex approach because it interacts with volume and total dose. In a fixed-dose trial, Zhai et al. compared 0.75%, 0.5%, and 0.25% ropivacaine by varying volume to keep the total milligram dose constant and found broadly comparable analgesia, with slower motor onset in the 0.25% group [10]. Xu et al. estimated the median effective analgesic concentration of 10 mL ropivacaine for arthroscopic rotator cuff repair at approximately 0.196% [13], but, as with dose-finding studies in other domains, such thresholds should not be generalized uncritically to all patient populations, surgical indications, or block techniques. A more recent double-blind trial further suggested that 7 mL of 0.1% ropivacaine may reduce hemidiaphragmatic dysfunction compared with the same volume of 0.5% ropivacaine while preserving postoperative analgesia [39]. Table 4 summarizes principal studies in this area and highlights how concentration, volume, and total dose are coupled in the existing literature.

### 8.4. Patient-Centered Implications

The rationale for reducing local anesthetic burden is intuitively strong from a patient-centered perspective. Lower concentrations or total doses may lessen the intensity or duration of motor block in some settings, potentially enabling earlier mobilization, greater respiratory comfort, and smoother discharge readiness, particularly in older patients or those with limited pulmonary reserve. However, these potential benefits must be demonstrated directly using outcomes that matter to patients, including pain at rest and with movement, opioid use, dyspnea and subjective respiratory comfort, diaphragmatic excursion, time to functional motor recovery, quality of recovery, and patient satisfaction.

### 8.5. The Gap That SWE May Address

Across the concentration-reduction literature, block efficacy and recovery have been evaluated primarily using clinical and patient-reported endpoints. These measures remain essential but do not describe tissue-level changes at the nerve or target muscle that may underlie different dosing strategies. SWE may help bridge this gap as an exploratory mechanistic endpoint, provided that acquisition protocols are standardized and that SWE findings are interpreted in parallel with clinically meaningful outcomes rather than in isolation. In this context, paired brachial plexus and deltoid SWE could be used in future studies to test whether lower-concentration regimens preserve effective analgesia while favorably shifting the balance between motor sparing, respiratory safety, and patient-centered recovery.

## 9. Integrating Shear Wave Elastography for Quantitative, Patient-Centered Interscalene Block

### 9.1. A Dual-Target Framework: Paired Nerve and Muscle SWE

The central proposition of this review is that paired nerve and muscle SWE could serve as a quantitative adjunct to conventional ISB assessment, capturing the local anatomical and mechanical changes that accompany injection and block development rather than the neurophysiological state of conduction blockade itself. Brachial plexus SWE may reflect nerve-level mechanical changes—volume effects, vasodilation, and local edema—that develop alongside, but are not synonymous with, sensory block. Anterior deltoid SWE may reflect target-muscle softening associated with reduced motor tone, which develops alongside, but is not equivalent to, motor block. At present, this should be regarded as a research framework rather than a validated clinical workflow.

Figure 4 summarizes the proposed dual-target concept. Brachial plexus SWE is positioned as an exploratory afferent or sensory-domain marker, while anterior deltoid SWE is positioned as an exploratory efferent or motor-domain marker. These quantitative measurements are not intended to replace clinical examination but to complement sensory testing, diaphragm assessment, motor recovery evaluation, and patient-reported outcomes [14,19,22,31] in both clinical research and, if validated, future practice.

### 9.2. SWE as a Tool for Concentration Optimization

The dual-target framework directly relates to the unresolved question of how best to optimize local anesthetic concentration, volume, and total dose while preserving analgesia and minimizing motor and respiratory burden. Future trials could compare lower- and conventional-concentration ISB regimens using a combined endpoint set that incorporates brachial plexus SWE, deltoid SWE, standardized sensory testing, motor recovery, diaphragmatic function, pain trajectories, opioid consumption, and patient-reported recovery. Such studies would begin to test whether lower-concentration strategies can maintain clinically effective block while improving outcomes that matter to patients, including mobility, breathing comfort, and satisfaction. Figure 5 provides a central visual summary of how paired nerve–muscle SWE could be embedded within this patient-centered optimization strategy.

### 9.3. Linking Quantitative Measurements to Patient-Centered Outcomes

The clinical value of SWE-defined block characterization ultimately depends on whether it improves decisions that are meaningful to patients. In the context of this Special Issue, the practical contribution of SWE would lie not in the elastography numbers themselves but in refining the “clinical art” of regional anesthesia—choosing techniques and doses that provide analgesia while minimizing respiratory discomfort, unwanted motor impairment, delayed mobilization, and dissatisfaction. For this reason, SWE studies should deliberately include validated patient-reported outcome measures, such as Quality of Recovery instruments and structured satisfaction scores, alongside objective respiratory and motor endpoints.

Table 5 outlines a suggested patient-centered outcome set for future ISB–SWE studies. Aligning SWE parameters with this type of outcome framework will be critical if quantitative imaging is to inform genuinely patient-centered regional anesthesia rather than remain a purely technical measurement.

### 9.4. A Practical Roadmap for Clinical Integration

Translation of this framework requires sequential validation rather than premature clinical adoption. First, acquisition protocols must be standardized for patient positioning, probe pressure, ROI placement, and acquisition timing. Second, the mechanistic basis of post-injection SWE signals must be characterized, distinguishing volume effects from pharmacological and secondary tissue changes. Third, SWE measurements must be correlated with established sensory, motor, and respiratory endpoints. Only after these prerequisites are met should prospective trials test whether SWE-informed dosing strategies add clinically meaningful value compared with current practice. Until such evidence is generated, SWE should be regarded as a promising research tool rather than a routine clinical requirement, and no decision algorithm should be proposed in clinical settings. Figure 6 outlines the open research questions corresponding to each of these sequential tiers.

## 10. Limitations of Current Evidence and Standardization Needs

### 10.1. Heterogeneity Across Studies

The current SWE literature relevant to ISB shows substantial methodological heterogeneity. Studies vary in equipment manufacturer and transducer specification, in ROI size and placement, in patient positioning, and in the timing of acquisitions relative to clinical events [13,19,20,21,22,23,24,27,31,32,33,34]. Reported normative values for the brachial plexus span a wider range than would be expected from biological variability alone, and direct numerical comparison across studies is consequently difficult. Until cross-platform calibration and consensus acquisition protocols have been established, conservative interpretation of absolute SWE values is warranted, and within-study or within-patient comparisons should be prioritized over between-study comparisons.

### 10.2. Reliability and Reproducibility

Inter- and intra-observer reliability for brachial plexus SWE has been formally evaluated in a small number of studies, with reported ICCs ranging from poor to moderate [21]. Reliability is generally better for the larger and more accessible peripheral nerves and for muscle [17,18,22], but the small cross-sectional dimensions of the cervical roots and the surrounding fascial complexity at the interscalene level make consistent ROI placement technically demanding. Operator training, structured acquisition protocols, and averaging of multiple measurements per ROI can mitigate, but cannot fully eliminate, this source of variability.

### 10.3. The Stress–Stiffness Confound

A persistent interpretive challenge is that SWE measures a composite of intrinsic tissue stiffness and applied tissue stress [30]. For nerves and muscle, the contribution of applied stress is non-negligible and can be modulated by limb position, respiration, and surrounding soft tissue tone. In the context of ISB, this means that part of any post-injection SWE change may reflect mechanical loading from the injected volume rather than the pharmacological effect of the local anesthetic. Disentangling these contributions experimentally requires controlled acquisition protocols with attention to volume, posture, and timing—factors that are not consistently reported in the existing literature.

### 10.4. Clinical Validation in Regional Anesthesia

Most published SWE studies of the brachial plexus and the deltoid have been conducted in healthy volunteers, in patients with non-anesthetic pathology, or in cadaveric and animal models [19,20,21,22,23,24,31,34,36]. Clinical validation specifically in the regional anesthesia setting—with paired pre- and post-block measurements, defined timing relative to block performance, and concurrent recording of clinical block characteristics—remains limited. Filling this gap is the immediate next step required for SWE to move from a research tool to a clinically actionable measurement.

### 10.5. Sample Sizes and Generalizability

Sample sizes in published SWE studies of the brachial plexus and deltoid are generally modest, often fewer than 50 participants, with limited representation of older adults, patients with comorbidities, and non-Western populations [20,21,24,33]. Generalization of normative values and reliability metrics to broader clinical populations is therefore tentative, and confirmatory studies in larger and more diverse cohorts are needed.

A critical limitation of the dual-target framework proposed in this review is that neither of its two components has been directly validated in the regional anesthesia setting. To our knowledge, no published study has yet validated brachial plexus SWE as a marker of intraoperative sensory blockade during interscalene block, nor has any published study validated anterior deltoid SWE as a marker of motor blockade induced by regional anesthesia. While each component is supported by indirect evidence—plexus SWE has been characterized in healthy volunteers and in chronic neurological disease, and deltoid SWE has been studied in reverse shoulder arthroplasty contexts—the capacity of either to capture block-related effects in real time has not been established empirically. The dual-target framework should therefore be understood explicitly as a research framework requiring prospective validation, not as a clinically deployable workflow. The causal correspondence between SWE values and the neurophysiological depth of block is itself unproven; observed post-injection stiffness changes likely reflect a composite of volume-related mechanical loading, vasodilation, local edema, and muscle relaxation, rather than a direct readout of sodium-channel blockade. SWE should therefore be evaluated as an exploratory surrogate of mechanical changes that accompany the block, rather than as a direct marker of sensory or motor block completeness.

A specific technical limitation applies to deltoid SWE in the regional anesthesia context. Healthy resting skeletal muscle already exhibits low baseline stiffness, and whether a further reduction in muscle stiffness produced by motor blockade is detectable as a statistically and clinically meaningful difference within the resolution and noise floor of current SWE platforms remains uncertain. A potential approach to this floor effect is to acquire SWE measurements under a defined, low level of passive stretch or load, which raises the dynamic range over which block-related softening can be detected; however, such standardized passive-loading protocols have not yet been established for the anterior deltoid in this setting. Future ISB-SWE studies will need to address measurement sensitivity, baseline variability, and standardization of loading conditions before deltoid SWE can be considered a reliable motor-domain endpoint.

## 11. Future Directions

### 11.1. Methodological Standardization

The most immediate priority for SWE in ISB research is methodological standardization. A consensus protocol should define patient positioning, transducer orientation, ROI dimensions, probe pressure minimization, and the number of repeated measurements, as well as the timing of acquisitions relative to block performance and clinical milestones. Standardized reporting of these parameters would facilitate comparison across studies, support meta-analytic synthesis, and clarify how much of the observed variability in SWE values reflects true biological differences versus technical factors [20,21].

### 11.2. Cross-Platform Calibration and Reference Ranges

A second requirement is cross-platform calibration. Current studies use different ultrasound vendors, transducers, and software implementations, which contributes to wide apparent variation in brachial plexus and deltoid stiffness values even among healthy volunteers. Multicenter efforts that acquire SWE data on the same participants using different systems, or that apply phantom-based calibration approaches, will be needed before robust, generalizable reference ranges can be established. Such work would also clarify whether device-specific correction factors are required for routine clinical or research use.

### 11.3. Time-Resolved, Mechanism-Informed Clinical Studies

Future clinical studies should be explicitly time-resolved and mechanism-informed. Rather than treating SWE as a single pre–post measurement, protocols could acquire serial nerve and muscle SWE values at predefined time points—baseline, early post-injection, during the block-onset window, and in the early postoperative period—while simultaneously tracking sensory changes, motor block, and diaphragmatic function. This approach would help disentangle volume-related mechanical loading from pharmacologic effects and edema, and would clarify which SWE trajectories, if any, correlate with the onset, quality, and resolution of block.

### 11.4. Integration with Automated Image Analysis

ROI placement and measurement averaging are operator-dependent steps that introduce variability into brachial plexus and deltoid SWE acquisition. Automated segmentation of these structures from B-mode and SWE images, using machine learning methods that have already been validated in other ultrasound applications, could reduce operator dependence and improve reproducibility. Embedding such automated analysis within commercial ultrasound platforms would also lower the technical barrier to adoption and make quantitative elastography more accessible in routine clinical settings.

### 11.5. Personalized Regional Anesthesia

A longer-term, currently speculative direction is the incorporation of baseline SWE into individualized regional anesthesia planning. Patients with altered nerve or muscle mechanical properties due to age, diabetes, prior radiation, or chronic shoulder pathology [24,33,34] may respond differently to a given local anesthetic regimen. Prospective studies that relate baseline plexus and deltoid stiffness to block characteristics, recovery trajectories, and complications could determine whether SWE-informed dosing or block selection genuinely improves patient outcomes. Population-scale validation would be essential before such personalized strategies could be recommended in clinical practice.

### 11.6. Patient-Reported Outcome Integration

Finally, future ISB–SWE studies should explicitly couple quantitative measurements with outcomes that patients can perceive and clinicians can act upon. These include movement-related pain, opioid consumption, dyspnea and respiratory comfort, time to functional motor recovery, readiness for discharge, Quality of Recovery scores, and structured satisfaction measures. As summarized in Table 5, aligning SWE parameters with a core patient-centered outcome set is essential if elastography is to inform the “clinical art” of regional anesthesia and optimize patient satisfaction, rather than remain a purely technical research tool.

### 11.7. Toward a Respiratory-Inclusive Multi-Target Framework

The dual-target framework as described focuses on nerve-level and target-muscle measurements (brachial plexus and anterior deltoid SWE). However, because hemidiaphragmatic paresis is the most clinically consequential adverse effect of ISB, a fully patient-centered evaluation framework should also incorporate quantitative assessment of respiratory function. Diaphragmatic ultrasound—measuring diaphragmatic excursion and thickening fraction during quiet breathing, deep inspiration, and the sniff maneuver—offers a non-invasive, real-time index of phrenic nerve function that is well-validated in critical care and is increasingly applied in regional anesthesia research [7,40]. A natural extension of the dual-target framework is therefore a triple-target paradigm in which paired brachial plexus and anterior deltoid SWE are acquired together with diaphragmatic ultrasound endpoints. By plotting time-resolved changes in nerve stiffness, target-muscle stiffness, and diaphragmatic excursion in parallel, future studies could quantitatively characterize the local anesthetic dose–response surface for the three principal trade-offs of ISB—analgesic efficacy, motor preservation, and respiratory safety—and identify candidate volume and concentration thresholds that optimize the balance between block efficacy and respiratory comfort. Direct SWE of the phrenic nerve is technically challenging because of nerve size and depth and is not a near-term clinical option, but diaphragmatic ultrasound provides a tractable indirect window onto phrenic nerve function and represents a methodologically realistic complement to nerve and muscle SWE in patient-centered ISB research.

## 12. Conclusions

Interscalene block remains a central regional analgesic technique for shoulder surgery, but its refinement requires a careful balance among analgesia, motor preservation, respiratory safety, and patient satisfaction. SWE offers a quantitative ultrasound-based method that may help characterize mechanical changes in the brachial plexus and deltoid muscle, yet the current evidence is preliminary and direct validation in the ISB setting is limited.

The dual-target framework outlined in this review—pairing brachial plexus and anterior deltoid SWE—should therefore be regarded as hypothesis-generating rather than as a prescriptive clinical algorithm. Future work should prioritize standardized acquisition protocols, paired nerve–muscle measurements, and rigorous correlation of SWE parameters with sensory, motor, and diaphragmatic endpoints, as well as with patient-reported recovery and satisfaction. If these efforts demonstrate added value, SWE could evolve into a useful adjunct for mechanism-informed, patient-centered regional anesthesia; until then, it should complement, rather than replace, clinical judgment and established outcome measures.

## Figures and Tables

**Table 1:** Normative Shear Wave Elastography Values for the Brachial Plexus at the Interscalene Groove.

Study	Population (*n*)	SWE Equipment	Measurement Site	Mean Stiffness (kPa, ±SD)	Reliability	Key Findings
Bedewi 2018 [20]	40 healthy adults (21 M, 19 F)	Aixplorer (SuperSonic)	C5, C6, C7 nerve roots	C5: 16.9 ± 4.9; C6: 15.7 ± 4.3; C7: 16.0 ± 4.6	Not reported	Sex differences at C6 and C7; no consistent age or BMI correlation
Aslan 2018 [21]	Healthy adults (pilot)	Aixplorer (SuperSonic)	C5–C7 roots, longitudinal and transverse	Reported per root and orientation; broadly overlapping with Bedewi	ICC: poor to moderate	Authors questioned current suitability of sonoelastography for routine BP assessment
Gürün 2022 [33]	32 MS patients vs. 32 controls	Aplio 500	C5, C6, C7 roots	MS group: elevated mean elasticity vs. controls; reduced root diameter	Good intra-observer	SWE detected subclinical demyelinating involvement

BMI, body mass index; BP, brachial plexus; ICC, intraclass correlation coefficient; MS, multiple sclerosis; SD, standard deviation; SWE, shear wave elastography.

**Table 2:** Factors Influencing Brachial Plexus Stiffness on SWE and Implications for ISB Study Design.

Domain	Specific Factor	Direction and Magnitude of Effect	Implication for ISB SWE Studies	Key References
Posture	Neck contralateral flexion	Increases brachial plexus stiffness; effect attributable to longitudinal nerve excursion	Standardize head and neck position throughout pre- and post-block acquisition	[28]
Posture	Shoulder abduction	Increases stiffness, particularly at C5–C6; magnitude varies with abduction angle	Maintain neutral or fixed shoulder position; document angle	[27,28]
Posture	Upper limb neurodynamic sequencing	Order of imposed joint movements alters timing and magnitude of stiffness change	Avoid pre-test mobilization; consistent positioning across patients	[27]
Patient	Sex	Significant differences at C6 and C7 (higher in males), small relative to inter-individual variability	Balance or stratify by sex in between-group comparisons	[20]
Patient	Age	No consistent association for brachial plexus; clear age-dependence for anterior deltoid	Stratify by age when deltoid is a paired endpoint	[20,24]
Patient	BMI and handedness	No consistent association reported; sample sizes limited	Record but not necessarily exclude	[20,21]
Comorbidity	Multiple sclerosis	Elevated mean elasticity; reduced nerve root diameter	Exclude or analyze as planned subgroup	[33]
Comorbidity	Post-radiation neural fibrosis	Stiffness elevation reported	Exclude prior thoracic radiation	[34]
Comorbidity	Diabetic polyneuropathy and chronic entrapment	Elevated nerve stiffness on SWE	Record diabetes and prior neuropathic conditions	[17,18,29]
Comorbidity	Chronic shoulder pathology	Not systematically evaluated; prevalence high among shoulder surgery candidates	Acknowledge as potential confounder	[23]
Measurement	Transducer compression	Light compression artifactually elevates stiffness	Minimize probe pressure; consider gel-pad or immersion	[28,32]
Measurement	ROI size and placement	Operator-dependent; small ROI variance contributes to poor-to-moderate ICC for BP	Define ROI a priori; average multiple measurements	[20,21]
Measurement	Probe alignment (longitudinal vs. transverse)	Anisotropy effect; longitudinal yields higher and more reproducible values	Standardize orientation; report consistently	[20,21,29]

BMI, body mass index; BP, brachial plexus; ICC, intraclass correlation coefficient; ISB, interscalene brachial plexus block; ROI, region of interest; SWE, shear wave elastography.

**Table 3:** Normative Shear Wave Elastography Values for the Deltoid Muscle.

Study	Population (*n*)	SWE Equipment	Measurement Site	Mean Stiffness (kPa, ±SD)	Reliability	Key Findings
Hatta 2016 [22]	8 fresh-frozen cadaveric shoulders	Aixplorer (SuperSonic)	Five deltoid segments, with passive elongation conditions	Reported per segment and position; values varied with passive elongation	ICC 0.761–0.963 (anterior, middle)	Reproducible SWE protocol established for deltoid; mechanical properties correlated with passive tension
Schmalzl 2022 [23]	Patients after reverse shoulder arthroplasty (*n* = 18)	Aixplorer (SuperSonic)	Anterior deltoid	Anterior deltoid stiffness correlated with patient-reported pain (VAS)	Not formally reported	Clinical correlation: higher anterior deltoid SWE associated with greater post-arthroplasty pain
Ivanov 2026 (online 2025) [24]	40 healthy volunteers (ages 20–80)	Aplio i800 (Canon)	Anterior, lateral, and posterior deltoid	Anterior: 23.2 ± 4.6; lateral: 26.4 ± 5.6; posterior: 17.9 ± 5.2	Moderate to good intra- and inter-rater	Significant age-dependent increase in anterior segment stiffness

ICC, intraclass correlation coefficient; SD, standard deviation; SWE, shear wave elastography; VAS, visual analog scale.

**Table 4:** Principal Studies of Local Anesthetic Concentration in Interscalene Brachial Plexus Block.

Study	Design	Concentrations Compared	Volume/Dose	Surgery	Key Findings
Klein 1998 [11]	RCT	0.5% bupivacaine vs. 0.5% ropivacaine vs. 0.75% ropivacaine	30 mL each	Shoulder	Comparable analgesia; ropivacaine showed slightly faster motor recovery
Riazi 2008 [9]	RCT	0.5% ropivacaine, 5 mL vs. 20 mL	25 mg vs. 100 mg	Shoulder arthroscopy	Equivalent analgesia; phrenic palsy reduced from ~100% to <50% with 5 mL
Borgeat 2010 [12]	RCT (continuous catheter)	0.2% vs. 0.3% ropivacaine infusion	Continuous, 48 h	Open rotator cuff repair	0.3% improved sleep quality and reduced morphine without increasing motor block
Lee 2011 [38]	RCT	0.75% ropivacaine, 5 mL vs. 10 mL	37.5 mg vs. 75 mg	Arthroscopic rotator cuff repair	5 mL provided similar analgesia with less phrenic paralysis
Zhai 2016 [10]	RCT	0.75% (6.7 mL) vs. 0.5% (10 mL) vs. 0.25% (20 mL)	Fixed 50 mg ropivacaine	Shoulder surgery	Comparable analgesia; 0.25% showed slower motor onset; same dose, different volume/concentration
Xu 2022 [13]	Up-and-down sequential allocation (*n* = 40)	0.1–0.35% ropivacaine	10 mL	Arthroscopic rotator cuff repair	MEAC = 0.196% (range 0.163–0.207% across four statistical models)
Verbeke 2026 [39]	RCT	0.1% vs. 0.5% ropivacaine	7 mL	Arthroscopic shoulder surgery	7 mL 0.1% ropivacaine reduced hemidiaphragmatic dysfunction versus 0.5%, but with potentially less effective postoperative analgesia

MEAC, median effective analgesic concentration; RCT, randomized controlled trial.

**Table 5:** Suggested Patient-Centered Outcome Set for Future ISB-SWE Studies.

Domain	Suggested Outcome	Rationale	Timing
Analgesia	Pain at rest and movement; opioid consumption	Preserves the core purpose of ISB	PACU, 6–24 h, and early rehabilitation
Motor recovery	Standardized shoulder/arm motor score; time to functional recovery	Captures motor-sparing benefit relevant to mobilization	Baseline, block onset, PACU, and follow-up
Respiratory safety	Diaphragmatic excursion or thickening fraction; dyspnea/respiratory comfort	Links phrenic involvement to patient experience	Baseline and 30–60 min after block
Recovery quality	Quality of Recovery score; discharge readiness	Connects mechanism-informed dosing with perioperative recovery	Postoperative day 0–1
Satisfaction	Structured patient satisfaction score	Reflects the special issue focus on patient-centered clinical art	Before discharge and/or follow-up

ISB, interscalene brachial plexus block; PACU, post-anesthesia care unit; SWE, shear wave elastography.

## Data Availability

No new data were created or analyzed in this study. Data sharing is not applicable to this article.

## Abbreviations

The following abbreviations are used in this manuscript:

Abbreviation	Definition
ARFI	Acoustic radiation force impulse
BMI	Body mass index
BP	Brachial plexus
ICC	Intraclass correlation coefficient
ISB	Interscalene brachial plexus block
LA	Local anesthetic
MEAC	Median effective analgesic concentration
MeSH	Medical Subject Headings
MS	Multiple sclerosis
NRS	Numerical rating scale
QST	Quantitative sensory testing
RCT	Randomized controlled trial
ROI	Region of interest
RSA	Reverse shoulder arthroplasty
SCM	Sternocleidomastoid
SD	Standard deviation
SWE	Shear wave elastography
SWV	Shear wave velocity
US	Ultrasound
